# Treatment challenges in pediatric Cushing’s disease: Review of the literature with particular emphasis on predictive factors for the disease recurrence

**DOI:** 10.1007/s12020-019-02036-2

**Published:** 2019-11-07

**Authors:** Katarzyna Pasternak-Pietrzak, Elżbieta Moszczyńska, Mieczysław Szalecki

**Affiliations:** 1 Department of Endocrinology and Diabetology, The Children’s Memorial Health Institute (CMHI), Al. Dzieci Polskich 20, 04–730 Warsaw, Poland; 2 The Medicine and Health Sciences Faculty, University of Jan Kochanowski, Al. IX Wieków Kielc 19A, 25-317 Kielce, Poland

**Keywords:** Cushing’s disease, Pituitary adenoma, Hypopituitarism, Adenomectomy, Transsphenoidal surgery

## Abstract

Cushing’s disease (CD) is a rare endocrine condition caused by a corticotroph pituitary tumor that produces adrenocorticotropic hormone. The current state of knowledge of CD treatment is presented in this article including factors that can be helpful in predicting remission and/or recurrence of the disease. The primary goals in CD treatment are quick diagnosis and effective, prompt treatment as the persistent disease is associated with increased morbidity and mortality. Cooperation of a team consisting of experienced pediatrician/adult endocrinologist, neuroradiologist, transsphenoidal neurosurgeon and (if necessary) radiotherapist contribute to the best treatment effects.

## Introduction

Cushing’s disease (CD) results from excess production of adrenocorticotropic hormone (ACTH) by pituitary adenoma [1]. The early intervention with effective treatment is crucial to prevent the consequences of long-term hypercortisolaemia [2]. The first-line treatment is pituitary surgery performed by transsphenoidal approach [3]. Remission rates in pediatric population vary depending on a center, neurosurgeon’s experience and other factors, and are reported to be between 45–95% [4–9]. In the case of relapse, second pituitary surgery or radiotherapy (RT) are considered. Pharmacological treatment is helpful in controlling hypercortisolemia during preparation for surgery or in case of refusal of surgical treatment and whilst awaiting the radiotherapy effects [10]. Herein, the current state of knowledge of CD treatment is presented together with description of predictive factors for CD recurrence. The article focuses on CD treatment of children and presents the comparison with the adult population highlighting the differences between these 2 groups of CD patients.

## Transsphenoidal surgery

Transsphenoidal surgery (TSS) is a first-line therapy of CD. The procedure is regarded as a safe and effective method as a selective microadenomectomy gives the possibility to preserve pituitary functions [3, 11, 12]. The technical difficulty of TSS results from the small sizes of ACTH-secreting adenomas and the pituitary fossa as well as from absent aeration of the sphenoid bone in young patients [13], hence the success of TSS depends on the surgeon’s expertize.

There are 2 different techniques of pituitary TSS: microscopic and endoscopic [14, 15]. The first one uses the standard microscopic transseptal approach popularized by Hardy in the late 1960s [14]. Thanks to microscopic three-dimensional visualization, operating in three-dimensional space is possible [15]. Endoscopic TSS, used first in adults since late 1990s, enables visualization of structures that are not in the direct line of vision (e.g., opticocarotid recesses, suprasellar areas) [16]. The drawback of this technique is two-dimensionality which impairs depth perception [16]. The endoscopic approach has given very good results in the treatment of pediatric patients with CD from the London center [17]: 5 of 6 patients were cured (remission rate 83.3%) and in 50% of them pituitary adenoma was not detected, while no tumor identification at TSS is regarded as poor predictor of remission rate [17]. Transcranial approach is no longer used as a primary technique and is performed only when the transsphenoidal approach is not possible or tumor size, its localization, and expansion requires this technique [2, 18, 19].

During the early weeks after TSS, neurosurgeons as well as endocrinologists should remember about possible complications that may occur such as: transient diabetes insipidus, hypopituitarism and, less frequently, syndrome of inappropriate antidiuretic hormone secretion or visual loss [20]. They are more often in the case of macroadenomas and extensive pituitary exploration [2, 19, 21]. Permanent pituitary dysfunction and diabetes insipidus are rare. Central hypothyroidism, GH deficiency or hypogonadism may also occur [20, 22]. The patients who require repeated transsphenoidal surgery are more likely to have complications such as: permanent diabetes insipidus, cerebrospinal fluid rhinorrhea, hemorrhage, injury of internal carotid, cranial nerve palsy and meningitis [23]. The mortality rate after TSS is low (<1%) [20, 24].

The loss of pituitary function depends on the aggressiveness of surgery; mean rates of hypopituitarism in adults range from 6.6% (selective adenomectomy) to 80.2% (total hypophysectomy) [1]. Post-TSS hypothyroidism occurs in 3.1–68.8%, hypogonadism in 0.6–46.1%, GH deficiency in 0.6–52.8% and panhypopituitarism in 1.4–29.3% of adult patients [1]. Data on pituitary function in long-term post-TSS assessment in children is limited comparing to data on adult patients. The examples of available limited pediatric literature review come from the centers in London and Bethesda [25, 26]. The London center provides data on the occurrence of long-term growth hormone deficiency (GHD) in 44% of patients (4/9 investigated), long-term hypogonadotropic hypogonadism (HH) in 19% of patients (all adults) and long-term panhypopituitarism in 19% of patients after effective surgical treatment (21 children, mean follow-up 10.6 yrs) [25]. For comparison, the National Institute of Health in Bethesda has published data based on much shorter mean time of follow-up (22 months) about the occurrence of secondary hypothyroidism in 16%, diabetes insipidus in 8%, GHD in 8% and HH in 3% of patients (50 children) [26].

## Cure of Cushing’s disease and predictors for remission

There is no consensus on the criteria for CD remission [2, 19, 21]. Different criteria used to establish CD remission or cure make the comparison of the treatment outcome in different studies difficult [1]. Furthermore, the results of studies evaluating mainly outcome of TSS cannot be adapted to different CD therapies [1]. Also the meaning of ‘remission’ of the disease is not unequivocal: there is temporary disease cure after TSS, when an apparent cure of the disease may be followed by a recurrence (which can be years or even decades after TSS) and a definitive disease cure when there is no relapse in long-term follow up [1].

### Postsurgical morning serum cortisol

Postsurgical morning serum cortisol is the most important marker to assess remission after TSS [27–29]. Recommended time when it should be measured varies depending on the center. Most authors suggest several measurements within 2 weeks after TSS, usually 5–14 days after surgery [21, 30, 31].

However, Atkinson et al. emphasize that the 0900 h serum cortisol may fall with time after surgery, sometimes over a few weeks [32]. For this reason other authors suggest that the serum cortisol measurement 1 to 6 months after TSS is better to establish the cure [33, 34]. Furthermore, mild or cyclical hypercortisolism may complicate the interpretation of TSS results [32].

Most studies in adults show long-term remission if postoperative (within 7 days after TSS) cortisol concentrations are <138 nmol/L (<5 μg/dL) [2, 19, 21]. The study by Trainer et al. gave the concept of more strict remission criteria: an undetectable (<1.8 μg/dl (50 nmol/liter)) postoperative 0900 h serum cortisol [35]. In this study in 20 of 48 adult patients after TSS for CD postoperative 0900 h serum cortisol was undetectable and in none of these patients the disease recurred (follow-up 40 months) [35]. Strict criteria are used more widely in children: serum cortisol <1.8 μg/dl (<50 nmol/l) at 0900 h within 2 weeks after surgery (cortisol measured 3–14 days after surgery) [13, 21, 30–32, 36]. It is important to withhold glucocorticoids treatment before cortisol measuring (min. 24-h break from the last dose of hydrocortisone (HC)) or to use low doses of dexamethasone (Dx) (<1 mg) [1].

It is noteworthy that no cortisol value (nor undetectable, nor subnormal) gives 100% certainty of no relapse [37, 38]. Alexandraki [28] has shown in the study of 131 adult CD patients that strict remission criteria were not superior in terms of the probability of recurrence compared with post-operative normocortisolaemia. Similarly, interesting conclusions can be drawn from the study by Lindsay et al. [37] of 331 patients (children and adults) with CD after TSS divided to 2 groups: group 1—patients with subnormal postoperative cortisol (2–4.9 μg/dl) and group 2—patients with postoperative cortisol < 2 μg/dl. It was hypothesized that patients with subnormal cortisol (group 1) might achieve long-term remission: the results showed that long-term remission rates were similar in 2 groups (2 μg/dl, 9.5%; 5 μg/dl, 10.4%; 2–4.9 μg/dl, 20%; not significant). Moreover, a cortisol serum value in the normal range predicts a recurrence in only ~17–31% [38].

According to the above, postoperative cortisol serum level alone is not an ideal marker of cure of CD and although the most important, it should be interpreted with other tests.

### Morning plasma ACTH

There is no sufficient data about the predictive value of postsurgical plasma ACTH levels for the disease remission. The Invitti et al. analysis on 288 adults has reported that only half of patients with clinical remission showed decreased plasma ACTH levels [39]. Contrary to the results in adults, in the retrospective study by Batista et al. (72 children with CD) children who remained in remission had significantly lower morning ACTH (and also cortisol levels) after TSS compared with those who relapsed (*P* < 0.001) [36].

### Urinary free cortisol

Urinary free cortisol (UFC) alone cannot be a measure of post-TSS remission, however, several UFCs levels below the normal range with concurrent evidence of disease remission in other tests can be useful to confirm disease cure [40]. Most studies in adults show long-term remission if postoperative UFC concentrations are <28–56 nmol/day (<10–20 μg/day) [2, 19, 21].

Studies on pediatric population show different results. In the Batista et al. study mentioned above, decreased UFC excretion after TSS was documented in all patients, including 4 patients with subsequent relapse of the disease, which confirms that UFC cannot be a predictive factor of long-term remission [36].

### Dexamethasone suppression test

Some authors tried to use LDDST as a predictor of remission (studies in mixed groups consisted of children and adults) but because of the use of different dosages and no agreement of the serum cortisol cut-off value this test is not used widely to establish the cure of CD [41–43]. LDDST as a predictive factor for remission in children only has not been tested.

### CRH stimulation test

Research in adults on the usefulness of CRH (corticotropine) test performed after TSS as a predictor of long-term remission was not conclusive. Avgerinos et al. analysis [44] has shown that there was no relapse in 23 adult patients who had a decreased response to CRH test performed after TSS (follow-up period 6–42 months after TSS). On the other hand, 3 patients (from 6 in this study), who had a normal response in the test, relapsed [44]. Lindsay et al. measured the response to oCRH (ovine CRH) in 331 children and adults (age at TSS 36 ± 0.8 yrs, range 5–71, follow-up 10.6 yrs) [37]. oCRH-stimulated cortisol (*P* < 0.002) and ACTH (*P* < 0.04) values were higher for the recurrence than the remission group. However, no basal or stimulated ACTH or serum or urine cortisol cutoff value predicted all who later recurred.

Contrary to the results of studies in adults/adults&children, in a study by Batista et al. (72 children, follow-up 24–120 months) a normal response to oCRH post-TSS was the predictive factor of nonremission [36].

Interestingly, Asuzu et al. used CRH stimulation test results to create new values named NEPVs (Normalized Early Postoperative Values) to improve prediction of nonremission [45]. NEPVs for cortisol and ACTH (calculated as immediate postoperative cortisol or ACTH levels minus preoperative post–CRH-stimulation test levels) predicted both early (10 days after TSS) and medium-term (11 months after TSS) nonremission (a group of adults&children) [45]. AUROC (area under the receiver operating characteristic curve) for NEPV of cortisol was 0.78 (95% CI: 0.61, 0.95); for NEPV of ACTH, it was 0.80 (95% CI: 0.61, 0.98), so these new values can be helpful in predicting nonremission after TSS for CD [45].

### Desmopressin stimulation test

In the early period after TSS, desmopressin (DDAVP) may stimulate ACTH secretion in the remnant corticotroph tumor (which have V3 receptors on their surface), but not in nontumor suppressed cells [46]. This fact can be used in the postoperative evaluation of cure. If the tumor is removed radically, ACTH and cortisol response to DDAVP disappears. Because, only 70–90% of patients with CD show an increase of ACTH and cortisol after DDAVP administration, the DDAVP test may be used only in patients with a positive response before surgery [47]. Furthermore, the Endocrine Society recommends its use as a part of research studies [48].

The results of two studies in adults/children & adults [49, 50] show usefulness of the desmopressin test in predicting CD recurrence. M. Losa et al. (the study on 107 adult patients) indicate that, during follow-up monitoring, three patients, who had persistence of the ACTH response to desmopressin, relapsed 24–54 months after TSS [49]. Barbetta et al. (the study on 68 patients aged 13–70 yrs) show that 3 patients (from 13 who relapsed) had positive responses to desmopressin which preceded the remission—they conclude that the positive responsiveness to desmopressin may be a criterion of risk for recurrence in patients who only normalized cortisol levels after surgery [50]. Unfortunately, there is no sufficient data on the usefulness of desmopressin test in children for prediction of CD relapse.

## Imaging

A study by Ciric et al. has shown that detection of adenoma in magnetic resonance imaging (MRI) is associated with a greater chance of tumor localization at surgery, and also gives a better prognosis for immediate postsurgical remission [51]. The remission rate after TSS (defined as undetectable serum cortisol in the immediate postoperative period <1.8 µg/dL) is reported from 45 to 95% [4–9]. Some data has shown that there are other factors decreasing immediate and long-term remission rates, such as: macroadenoma, dural or cavernous sinus invasion, postoperative eucortisolism (in the absence of preoperative or postoperative medical treatment) and absence of tumor on MRI or ACTH positive tumor in pathology [2, 52].

Because the size of pituitary adenomas detected in MRI in children is usually less than 6 mm and their visualization rate is relatively poor due to the limited spatial resolution of MRI [6], there was a necessity of creating better imaging techniques. Spoiled gradient-recalled acquisition in the steady-state (SPGR) with improved spatial resolution is considered better than the conventional T-1 weighted spin echo (SE) technique in identifying pituitary tumors. The sensitivity of SPGR in detecting microadenomas is 80% in adults (vs sensitivity of T1 spin echo imaging 49%) and 68% in children (vs. sensitivity of T1 spin echo imaging 29%) [53, 54]. Comparably, detection of microadenomas is improved by demonstrating microadenomas as hypo-enhancing regions using volumetric gradient recalled echo (3D-GRE) [53, 55]. Using the fact that contrast may be retained longer in pituitary adenomas compared with normal pituitary gland, delayed postcontrast FLAIR (fluid attenuated inversion recovery) imaging may have utility in identifying delayed contrast retention in pituitary adenomas [56, 57]. The Chatain et al. study suggests that delayed microadenoma contrast washout may be detected as FLAIR hyperintensity in otherwise MRI-negative CD cases [58]. The authors of this study propose adding postcontrast FLAIR sequences to complement 3D-GRE for surgical planning in patients with CD [58]. Different technique—18F-FDG PET/CT, is not recommended to diagnose CD. Feng et al. reported that the sensitivity of 18F-FDG PET was 67% and even lower for patients after treatment, based on analyzed 43 CD patients [59]. Zhoua et al. in their study proved that 18F-FDG PET/CT plays a role in localizing the site for ectopic ACTH-dependent syndromes, although it plays a limited role in CD [60].

## Predictors of recurrence

Although many researchers tried to find predictors for CD relapse, the results differ upon the study and, unfortunately, there is no universal prognostic factor that would be confirmed in all studies. Factors that can be associated with the increased risk of CD recurrence in adults and in children are presented in Table 1.

**Table 1:** Predictors of CD recurrence in adults and children

Predictors of recurrence in adults	Predictors of recurrence in children
• male gender	
• younger age at diagnosis	• older age at the time of disease symptoms
• longer duration of symptoms	• younger age at the time of surgery
• severe clinical presentation	
• depression or behavioral symptoms	
• significantly elevated serum cortisol and UFC levels preoperatively	
• higher postoperative basal and oCRH-stimulated cortisol/ACTH levels^a^	
• no evidence of adenoma in MRI	
• no tumor identification at surgical intervention	
• no tumor identification at post-surgical pathological examination	
• large macroadenomas (tumor diameter ≥ 2,0 cm)	• larger tumor diameter
• tumor extension, especially to the suprasellar region and with the involvement of the pituitary intermediate lobe	
• dural invasion or petrosal sinus invasion	
• an early recovery of HPAA after TSS	
• no histopathological confirmation of the pituitary corticotroph adenoma	
• the absence of peritumoral Crooke’s cells	
	• mutations in *USP8* gene in resected tumor tissue^a^
[28, 29, 33, 41, 42, 52, 61–65, 69, 70, 112–121]	[7, 8, 30, 36, 61, 66, 67, 114]

^a^explanation in the text

Several studies on adults assessing the usefulness of oCRH test in predicting CD recurrence have shown that the relapsing patients had higher cortisol or ACTH responses to oCRH than patients who stayed in remission [44, 61–64]. In the Alwani et al. study on 79 adults the absolute peak cortisol concentration after oCRH test gave the best diagnostic accuracy in predicting outcome of cortisol cutoff value of 600 nmol/l) [62]. Baseline plasma ACTH levels and peak cortisol responses to oCRH were the best parameters for predicting relapse after TSS in the Invitti et al. study [63]. This study confirmed the usefulness of post-TSS CRH testing, because recurrence developed only in patients presenting a response of both hormones to CRH stimulation [63]. Comparably, in Lindsay et al. study mean basal and stimulated ACTH and stimulated cortisol values were significantly lower for patients in long-term remission compared with those who later recurred (*p* < 0.007–0.02) [37].

In the Alexandraki et al. study of 131 adults with CD the time of hypothalamic-pituitary-adrenal axis (HPAA) recovery was the only prognostic factor for CD recurrence [28]. Chun-Heng Kuo et al. (a group of 52 adults) have shown that ACTH level before treatment had the positive correlation with the recurrence of the disease [65].

As CD is rare in childhood, there is very limited data (in comparison to data on adult patients) with extended follow-up reporting prognostic factors of the disease recurrence in this population. The analysis by Lonser et al. of 200 children with CD (mean postoperative follow-up 6.8 ± 4.7 yrs, range 0.3–21.3) has shown the following prognostic factors of CD recurrence: dural invasion or petrosal sinus invasion, older age at the time of disease symptoms (in contrast to younger in adults), no identification of adenoma during surgery and larger tumor diameter [8]. In the analysis by Devoe et al. of 42 children (mean follow-up 7.2 yrs, range 1.5–13.6) with CD recurrence of the disease was correlated with a younger age at the time of surgery [7]. Lodish et al. [30] have shown that an early recovery of HPAA after TSS may indicate disease recurrence (57 children, follow-up 6–36 months). A study by Batista et al. (72 children, follow-up 24–120 months) has shown the following factors associated with relapse: lack of histological confirmation of an adenoma, higher post-TSS serum cortisol or ACTH levels, a higher ACTH and cortisol responses to oCRH (CRH test performed after TSS), and glucocorticoid replacement for less than 6 months after surgery [36]. Also post-TSS morning cortisol and ACTH value mentioned above as predictors of long-term remission can be helpful in predicting recurrence of the disease.

Recent studies, both on pediatric (Faucz et al.; 42 children) and adult population (Albani et al.; 48 adults), indicate that recurrences appear to be more frequent in patients with *USP8* mutant corticotroph tumors [66, 67]. However, these results are in contrast to the previous reports in adults (Hayashi et al., 60 adults) suggested that the *USP8* mutated tumors are not as aggressive and that the long-term remission rates in patients with detected *USP8* mutation are higher [68].

The recurrence rates are reported in 6–27% children after initial remission [4, 8, 36] and these results differ from the recurrence rates in adults who more often relapse—3 to 47% [37, 48, 69]. CD recurrence was documented even after 15 years of successful surgery (in adult patient) [28], hence long-term follow-up of patients after TSS is crucial. In contrast to presented above data about lower recurrence rates in children, results of Leinung et al. study indicate that children and adolescents with CD are at greater risk of relapse than adults [4].

## Treatment in case of the disease recurrence or lack of remission

The options of treatment for patients who do not achieve remission after TSS are: second pituitary surgery, pituitary radiotherapy, long-term medical therapy to control hypercortisolemia and bilateral adrenalectomy (BA) detailed below. In subjects with uncured/recurrent CD, treatment options must be individualized.

### Second pituitary surgery

Second pituitary surgery is a good option when residual tumor is well visualized in MRI or has regrown but is not invasive [2, 19, 21]. Resection success rates (in adults) are lower in comparison to the first TSS (50–73% vs 81%) [70].

### Pituitary radiotherapy

Pituitary radiotherapy is a good first-line treatment when the surgery cannot be performed or a second-line approach in the case of persistent disease/recurrence after surgery, especially when the tumor is invasive [2, 19, 21]. Conventional fractionated external beam radiotherapy delivers dosage of 4500–5000 cGy total, and is usually given in 180–200 rad fractions over a period of 6 weeks [20]. Intensity-modulated radiotherapy (IMRT) enables dose adjustment for tumor contours and spares nearby crucial structures. There are newer forms of RT available now: stereotactic RT, photon knife (computer-assisted linear accelerator) and the gamma knife (cobalt–60).

From available literature (Table 2), mean time to cure in adults is 1.5–5 years [71, 72] and the cure rates of conventional fractionated RT are 56–83% [71, 72]. Despite the published data of the results in children are limited, available date provide that the mean time to cure in children is shorter: 0.75–2.86 years and that the cure rates are higher in comparison to adults—50–100% [5, 10, 73–75]. According to studies in adults (by Schteingart) and children (by Jennings), there are some promising results of a combined pituitary RT and mitotane, which improves the success rate of either modality given alone curing ~66% patients [74, 76].

**Table 2:** Outcome of pituitary radiotherapy in children

Series	RT	No CD patients treated by RT	Cure rate	Time to cure	Follow-up	Pituitary function at the time of last follow up
Storr [10]	45 Gy, in 25 fractions	7	7/7 (100%)	0,94 yrs (0,25-2,86)	6.9 yrs	GHD in 33% (6/18) patients (follow-up 0.6–2.5 yrs) At 2 yrs post RT, puberty occurred early in one male patient (age, 9.8 yrs); normal in the others. Serum T4, TSH, and PRL levels within the normal reference ranges throughout follow-up
Acharya [5]	45 Gy in 25 fractions	8	4/8 (50%)	9-18 mos.	2 yrs	5 patients were hypogonadal; 1 patient was hypothyroid. All patients were below their target height at the time of last follow up. None of the patients had posterior pituitary dysfunction
Jennings [74]	45 Gy in 25 fractions	15	12/15 (80%)	1-18 mos.	1–18.25 yrs	Growth resumed in 12; Sexual development proceeded normally in all 15
Thoren [75]	stereotactic external RT using 60 Co gamma radiation (dose 50-70 Gy)	8	7/8 (87,5%)		2.6–6.75 yrs	GHD in all 2/8 p were given thyroxine substitution in 3/8 secondary hypogonadism
Chan [73]	45 Gy in 25 fractions	12* (*long term data in 6/12 patients)	11/12 (92%)	0.13–2.86 yrs	6.6–16.5 yrs; mean 10.5 yrs	At a mean of 1.0 year (0.11–2.54) following RT, GHD in 5/6 patients. On retesting at a mean of 9.3 years (7.6–11.3) after RT, three out of four patients were GH sufficient (peak GH 19.2–50.4 mU/l). Other anterior pituitary functions including serum prolactin in five out of six patients were normal on follow-up

The outcome of pituitary irradiation in CD has been reported in a number of small studies that focus mainly on adult population. Hypopituitarism is the most common adverse effect of RT, more frequent if TSS is performed before RT [24]. The other adverse effects of RT (visual impairment, radiation oncogenesis) are very rare [77, 78]. Estrada et al. in his analysis of 30 adult patients with persistent or recurrent CD have shown that 57% of patients (17/30) had GHD after RT (GHD was diagnosed if plasma growth values were <15 mIU/L after the inducement of hypoglycemia), 33% (10/30) had gonadotropin deficiency, 13% (4/30) had a deficiency of thyrotropin, and 3% (1/30) had a deficiency of corticotropin [77]. 83% had remissions during a median follow-up of 42 months (range: 18–114). The remissions began 6–60 months after RT, but in most cases (73%) remission occurred during the first 2 years. None of the patients had a relapse of CD after remission was achieved [79].

Results from studies performed on pediatric population present that in children after RT pituitary deficiencies do not occur so often. Storr et al. described the efficacy of RT in 7 children treated by pituitary RT post-TSS [10]. GH secretion was assessed at 0.6–2.5 yrs post RT in all patients: 33% (6/18) of patients had GHD (GHD was defined as peak GH <20 mIU/L). At 2 yrs post RT, puberty occurred early in one male patient (age 9.8 yrs) but was normal in the others [10]. Serum T4, TSH, and PRL levels remained within the normal reference ranges throughout mean 6.9 yrs follow-up. Similarly, Chan et al. reported in their retrospective analysis the results of anterior pituitary function in 6 patients treated with pituitary RT after >6 yrs follow-up [73]. Despite the fact that at a mean of 1.0 year (0.11–2.54) following RT, GHD was present in 83% patients (GHD in childhood was defined as peak GH on provocation testing <20 mIU/L; severe GHD in pediatric and adult patients was defined as peak GH level <9 mIU/l), on retesting at a mean of 9.3 yrs (7.6–11.3) after RT 75% patients were GH sufficient. Other anterior pituitary functions including serum PRL in 5/6 patients were normal on follow-up [73].

### Pharmacotherapy

Definitive treatment such as TSS (and RT in the case off TSS failure), rather than pharmacotherapy, is currently recommended for the management of pediatric CD. Drug therapies for children with CD are limited and not well studied. They can be applied to urgently lower cortisol level in patients with severe hypercortisolemia in preparation for surgery or whilst awaiting the effects of RT [10]. Long-term treatment may not be effective because of oversecretion of corticotrophin [2].

There are several drugs that can be used: Ketoconazole and other adrenal enzyme inhibitors: metyrapone, aminoglutethimide and trilostane may be used alone or in combinations to control hypercortisolism but they do not destroy adrenocortical cells that secrete cortisol [20]. None of these drugs is approved by the FDA (US Food and Drug Administration) for CD treatment [80]. Ketoconazole is an agent that affects many P450 enzymes (depending on the dose used) blocking adrenal steroidogenesis. Ketoconazole therapy requires liver function monitoring [81]. The dose is 300 to 1200 mg/day [81, 82] and 45–50% of patients show long-term control with continued use [81]. Ketoconazole has not been approved by the FDA for CD treatment neither in children nor in adults [80], because of the risk of severe liver injury and harmful interactions with other medications [80]. The European Agency EMA had recommended a permission of a marketing authorization for KCN (HRA Pharma) in the treatment of CS [83]. Metyrapone increases cortisol metabolites in the serum and urine due to the predominant inhibition of 11-hydroxylase (also the other steroidogenesis enzymes but to a lesser extent) [84]. Metyrapone has been used safely in children awaiting for RT results [10], the recommended dosage is 750–2250 mg/day [81]. Metyrapone is approved for the treatment of CS in the European Union in adults. Mitotane decreases cortisol by direct inhibition of steroidogenesis at a few enzymatic steps [85]. It also destroys adrenocortical cells secreting cortisol. Therapy with mitotane alone can be successful in up to 72% of patients with CD [86]. Mitotane (3 mg/day) can be used also as an additional drug with metyrapone [10]. Aminoglutethimide blocks the conversion of cholesterol to pregnenolone in the adrenal cortex, inhibiting the synthesis of cortisol, aldosterone, and androgens. Aminoglutethimide can be used in a combine therapy with metyrapone (the dosage is 1 g/day) [10]. Trilostane inhibits the conversion of pregnenolone to progesterone. Storr et al. described in their study successful use of oral drugs (ketoconazole, metyrapone, aminoglutethimide or mitotane) to control hypercortisolemia in 8 patients after pituitary RT [10].

Another pharmaceutical drug—etomidate has been occasionally used in the treatment of CD [87, 88]. Greening et al. described a 6.2-year-old male patient with severe hypercortisolemia and life-threatening complications (respiratory failure, severe psychosis) of CD in whom the therapy with metyrapone and ketoconazole was ineffective. Intravenous administration of etomidate had successfully lowered the cortisol level before bilateral adrenalectomy was done [88].

There are also newer therapies for patients unsuccessfully treated by surgery that directly affect the pituitary tumor: cabergoline and pasireotide [89, 90]. Cabergoline is a dopamine agonist and its role in CD treatment has been debated. Pivonello in his study [89] has shown that cabergoline treatment is effective in controlling cortisol secretion for at least 1–2 yrs in more than 33% of a limited population of patients with CD (20 patients in the age 24–60 yrs with persistent CD after unsuccessful surgery). Currently, cabergoline is not approved by FDA for CD treatment.

Specific somatostatin analogs are promising in achieving CD therapeutic goals. Tumors corticotroph cells have on their surface somatostatin receptors (SSTR), mainly SSTR5 and SSTR2 [91–93]. The SSTR subtype 5 has become a therapeutic target in patients with CD thanks to the use of the somatostatin analog—pasireotide that has the highest affinity for this receptor subtype [89, 90, 94]. Both subtypes: SSTR5 and SSTR2 have been shown to be involved in the regulation of ACTH release [86, 95]. Several studies (including large phase III clinical trial) have proven that pasireotide causes normalization of urinary cortisol in 25–30% of CD adult patients [90, 93, 96, 97]. Pasireotide (Signifor) has been approved for adult CD treatment by the FDA and the European Commission [80]. The use of pasireotide in children is limited to single cases and there are no studies summarizing the effects of treatment in this group of patients. Yordanova et al. describes 1 female patient diagnosed with CD at the age of 13.8 yrs, who had relapse of CD 6 years after TSS [25]. The patient refused BA and was being managed with pasireotide with good control of hypercortisolemia.

Recently, mifepristone (a progesterone receptor antagonist with glucocorticoid receptor antagonist activity at higher doses) was approved by FDA for treatment of adults with CS to control hyperglycemia [98]. Improvement in clinical, metabolic, and psychosocial outcome in adults has been documented [97]. Data on the use of medical therapies to treat CS in children and adolescents are limited [99]. At present, mifepristone is the promising option for long-term medical treatment of refractory CD in children. Unfortunately the clinical trial “Mifepristone in children with refractory Cushing’s disease”, which was to be the largest study on the effects of mifepristone in children gave no results because of lack of patients enrollment.

### Bilateral adrenalectomy

Bilateral adrenalectomy is the treatment option for CD patients who have either failed surgery or RT or where TSS is not possible or available. If performed properly, BA provides immediate relief from hypercortisolism. A laparoscopic approach is the preferred method as is associated with reduced morbidity. There are some disadvantages of BA for the patients: 1. a necessity of lifelong replacement with glucocorticoids and mineralocorticoids; 2. BA does not eliminate the cause underlying the hypersecretion of ACTH; 3. the perioperative mortality is approximately 3 times higher than that of TSS (3% vs. 1% in TSS); 4. recurrences can (rarely) occur (as the result of the growth of adrenal rest tissues) [20]; 5. the risk of Nelson’s syndrome (NS)—an important complication of BA when the patient develops macroadenomas that secrete ACTH [100].

NS was documented months or years in up to 15% of children with CD after BA [20, 24]. NS appears to be more frequent in children than in adults (an incidence of 8–43% in adults [101] and 25–75% in children [102–104] and often requires pituitary surgery or RT [105]. Children after BA seem to have higher risk of NS in comparison to adults—for this reason they should be carefully monitored—they require annual monitoring with MRI and assessment of plasma ACTH values [102–104].

## Quality of life

Numerous studies assessing the quality of life in patients after CD treatment have been published, but they mainly concern adults [106, 107]. Available data had shown that adult CD patients still experience significant problems in many areas of life, even if they achieved biochemical remission and are many years after definitive treatment. CD-induced hypercortisolemia, even cured, may have long-term adverse effects affecting mood and functioning in society [106]. Van Aken in his analysis of 58 adults after an average of 13.4 yrs of follow-up underlines that patients general QoL is decreased, with mainly weakened psychosocial and physical spheres, especially in those with hypopituitarism [107]. It has also been proven that CD patients have impaired psychological well-being and psychosocial functioning compared to patients with other pituitary tumors [106, 108–110].

Similar conclusions are provided by the only prospective study by Keil et al. conducted on pediatric population [111]. The study (40 children with CS including 34 children with CD) documented that pediatric CS is associated with impaired HRQL one-year after cure [111]. Post-TSS scores of all CS patients remained significantly below US normative data for: physical function (*P* < 0.02), role-physical (*P* < 0.02), global health perception (*P* < 0.001), emotional impact (parent) (*P* < 0.001) and physical summary score (*P* < 0.001) [111]. The findings of these studies demonstrate that children and adolescents affected by CS have impaired QoL, which only partially resolves after successful treatment.

## Abbreviations

ACTH: adrenocorticotropin
CD: Cushing’s disease
CRH: corticotrophin
CS: Cushing’s syndrome
HPAA: hypothalamic-pituitary-adrenal axis
min.: minimum
mos.: months
MRI: magnetic resonance imaging
o: ovine
RT: radiotherapy
TSS: transsphenoidal surgery
yrs: years
